# Towards causal inference-based antidepressant selection with brain and blood biomarkers

**DOI:** 10.1038/s41386-025-02183-3

**Published:** 2025-09-05

**Authors:** Milica Barac, Caroline W. Grant, Russell Toll, Thomas Carmody, Abu Minhajuddin, Cherise Chin Fatt, Jane A. Foster, Paul E. Croarkin, William V. Bobo, Manish K. Jha, Arjun P. Athreya, Madhukar H. Trivedi

**Affiliations:** 1 Department of Molecular Pharmacology and Experimental Therapeutics, Mayo Clinic, Rochester, MN USA; 2 Center for Depression Research and Clinical Care, Peter O’Donnell Jr. Brain Institute and the Department of Psychiatry, University of Texas Southwestern Medical Center, Dallas, TX USA; 3 Department of Health Data Science and Biostatistics, Peter O’Donnell Jr. School of Public Health, University of Texas Southwestern Medical Center, Dallas, TX USA; 4 Department of Psychiatry and Psychology, Mayo Clinic, Rochester, MN USA; 5 Department of Behavioral Sciences and Social Medicine, Florida State University, Tallahassee, FL USA

**Keywords:** Depression, Predictive markers

## Abstract

This report sought to employ multi-modal integration of pre-treatment brain (electroencephalogram, resting-state functional magnetic resonance imaging) and blood (immune and metabolic) biomarkers to facilitate causal inference-based treatment selection by virtue of establishing predictability of remission to multi-stage antidepressant treatment. Data from two stages of pharmacotherapy in the ‘Establishing Moderators and Biosignatures of Antidepressant Response for Clinical Care for Depression’ (EMBARC) study from participants with both brain and blood biomarkers were included (*N* = 197). Participants were initially randomized to sertraline or placebo (Stage 1), and depending on clinical response at week-8, their therapy in Stage 2 was either maintained or switched (to sertraline, if a non-responder to placebo, or to bupropion, if a non-responder to sertraline). Three readily accessible clinical features combined with 15 multi-modal features associated with baseline depression severity predicted stage 2 remission with an AUC of 0.74, 0.71, and 0.73 for sertraline, bupropion, and placebo treatment respectively. Propensity score-matching (causal inference) was conducted across Stage 2 treatment arms, and the same features were used to build an unsupervised model to produce the probability of remission to the given Stage 2 treatment (as factual outcome), as well as the alternative treatment not given (as counter factual). While the accuracy of observed outcomes across treatment arms was 82%, the accuracies of predicted counterfactual (unobserved) outcomes warrant future prospective studies. 16 weeks and associated biomarker-based prediction of counterfactuals suggest that the selected markers are highly sensitive features for guiding antidepressant treatment selection.

## Introduction

Major depressive disorder (MDD) is the leading cause of disability worldwide [1, 2]. Only a third of patients achieve remission from first-line antidepressant therapies [3], with treatment failures adding to disease and economic burden [4]. Given heterogeneity in disease symptom severity and response trajectories, there is keen interest in precision medicine approaches for treating MDD and individualizing the selection of antidepressants by utilizing prognostic biomarkers and or biosignatures of disease pathophysiology [5].

Two primary modalities of biomarkers have been studied thus far for establishing predictability of antidepressant response, namely, blood (metabolomics [6], genomics [7], immune-proteomics [8]) and brain (magnetic resonance imaging (MRI) [9], electroencephalographic (EEG) [10]) biomarkers. Machine learning (ML) approaches utilizing these biomarkers as predictors of antidepressant (escitalopram, bupropion) response at 8 or 12 weeks achieved area under the receiver-operating curve (AUC) of 0.64 to 0.86 [11, 12]. Multimodal integration of functional MRI (fMRI) and blood biomarkers has demonstrated improved performance compared to single modality models [13]. In a review predicting MDD treatment outcomes using ML methods, only one used a combination of predictors from three modalities (clinical, cognitive, and neuroimaging) [14]. Thus, there continues to be a knowledge gap on whether the integration of blood and brain derived biomarkers could (a) improve predictability of sequential treatment outcomes over a single modality, and (b) aid in treatment selection.

With the goal of elucidating blood or brain biomarkers that could predict multi-stage remission (i.e., 2 or more successive pharmacological treatments for MDD) and eventually facilitate treatment selection, the Establishing Moderators and Biosignatures of Antidepressant Response in Clinical Care (EMBARC) study collected both brain and blood measurements [15]. The EMBARC study was a multisite double-blinded randomized clinical trial designed to identify potential biomarkers of antidepressant response [15]. In one prior report, a model using clinical features and pretreatment theta current density, obtained from electroencephalogram (EEG) data, predicted placebo responders and remitters at week 8 with an AUC of 0.73 and 0.76 respectively [16]. Another report using fMRI data from the EMBARC study predicted response to sertraline at week 8 with an AUC of 0.73 [17]. While prior reports using EMBARC data investigated response or remission outcomes at week 8, no study from EMBARC to date has (a) integrated blood and brain biomarkers to predict treatment outcomes, and (b) identified pre-treatment biomarkers that can facilitate treatment selection based on prediction of remission (or the lack of) at 16 weeks.

**Fig. 1:**
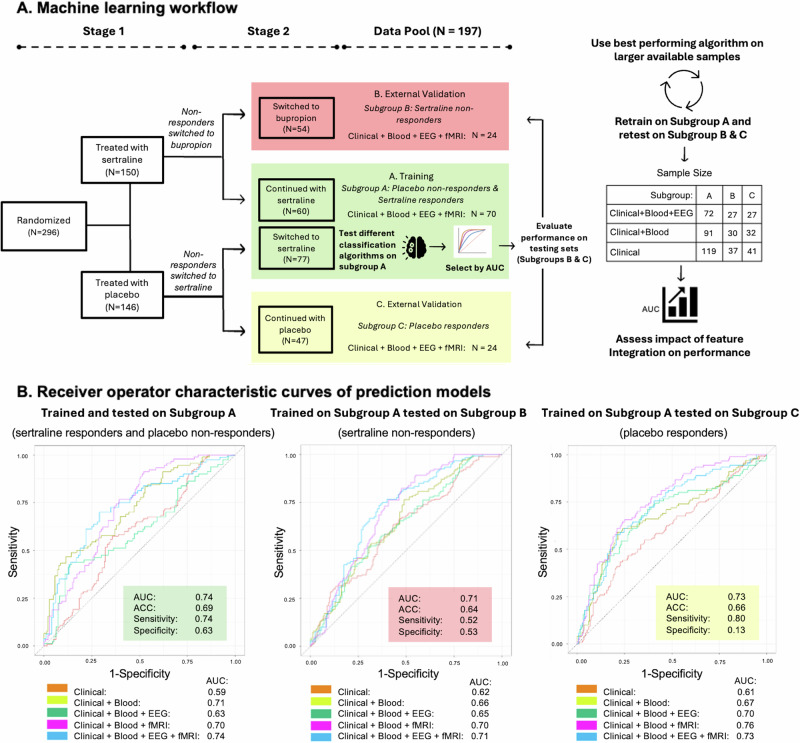
Study breakdown of EMBARC with machine learning workflow and results. **A** Breakdown of the study arms of EMBARC, as well as the classification of Subgroups A, B, and C is displayed. The workflow of training the model on Subgroup A and testing on Subgroup B & C is repeated on several samples with varying levels of data completeness. **B** Receiver operator characteristic curves of training and testing sets. ACC: Accuracy. AUC: Area Under the Curve.

The goal of this report (see Fig. 1A for overview) was to employ multi-modal integration of pre-treatment brain and blood biomarkers to facilitate treatment selection (using causal inference) by establishing predictability of remission to multi-stage antidepressant therapy (using supervised ML). The analytical emphasis was on answering the question (referred to as the counterfactual hypothesis in this work), “if a patient was a non-remitter to a treatment “x”, what was the likelihood of remission to the alternative treatment “y”, based on factual outcomes of participants with similar biological profiles (e.g., similar EEG, fMRI, immune profiles) who were given the alternative treatment “y”. In this work’s context, the counterfactual is the hypothetical scenario that explores the likely outcome had the patient been treated with an alternative antidepressant. The overarching hypothesis of this work is that brain and blood biomarkers associated with depression severity at baseline would facilitate the prediction of Stage 2 remission: thereby generating the counterfactual hypothesis regarding the likelihood of remission to the other antidepressant treatment.

## Materials and methods

### Study design and population

This is a secondary analysis of data from the EMBARC study (clinicaltrials.gov ID: NCT01407094) [15]. Briefly, the EMBARC study was a multisite double-blind randomized clinical trial consisting of 296 outpatients diagnosed with nonpsychotic MDD, designed to identify pretreatment and early-treatment (week 1) clinical, neuroimaging, and blood moderators for predicting response to sertraline or placebo. The EMBARC trial was approved by the Institutional Review Board at each clinical site, and all patients provided informed consent upon study entry. Demographic information and clinical characteristics of participants is provided in Table 1. The EMBARC study comprised two stages; in Stage 1, the first 8 weeks of the study, all participants were randomized to either placebo (*N* = 150) or sertraline (*N* = 146). At week 8 (end of Stage 1), participants were assessed with the Clinical Global Improvement scale (CGI), and those receiving a score of less than “much improved” were considered non-responders [18]. In Stage 2, placebo non-responders switched to sertraline, and sertraline non-responders switched to bupropion. Responders at Stage 1 continued with their previously assigned treatment plan.

**Table 1:** Demographic characteristics by treatment arm.

	Placebo (*N* = 150)	Sertraline (*N* = 146)	Total (*N* = 296)
**Age**			
Mean (SD)	36.9 (12.8)	37.2 (13.8)	37.1 (13.3)
Median [Min, Max]	35.5 [18.0, 65.0]	33.0 [18.0, 65.0]	34.5 [18.0, 65.0]
**Sex**			
Female	92 (61.3%)	102 (69.9%)	194 (65.5%)
Male	58 (38.7%)	44 (30.1%)	102 (34.5%)
**Race**			
American Indian or Alaska Native	1 (0.7%)	0 (0%)	1 (0.3%)
Asian	13 (8.7%)	8 (5.5%)	21 (7.1%)
Black	25 (16.7%)	33 (22.6%)	58 (19.6%)
Native Hawaiian	0 (0%)	0 (0%)	0 (0%)
White	103 (68.7%)	90 (61.6%)	193 (65.2%)
Other	8 (5.3%)	15 (10.3%)	23 (7.8%)

### Primary outcome measure

The primary measure of outcome assessed of the EMBARC study was symptom severity measured using the Hamilton Depression Rating Scale (HAM-D) [19]. Remission at week 16 was defined as a 17-item HAM-D total score ≤ 7 [20].

### Brain and blood measures

#### Functional magnetic resonance imaging (fMRI)

MRI scans were collected on 3 Tesla MR systems at all EMBARC sites. Briefly, the data were preprocessed with a standard pipeline, namely slice time correction, motion corrected (realignment and unwarp), spatial normalization, and smooth with an 8 mm Gaussian Kernel. Complete fMRI preprocessing is listed in Supplementary Materials 1.

#### Electroencephalography (EEG)

As described in previous studies, different systems were used at each site to acquire high-density EEG (60 to 128 electrodes) [21], and harmonized using established procedures [22]. Additional details of EEG preprocessing are in Supplementary Materials 1.

#### Blood markers

Peripheral blood samples were collected in EDTA tubes at all EMBARC sites. The complete list of analytes measured, and data processing details are available in Supplementary Materials 1, Supplementary Tables 1 and 2, and Supplementary Fig. 1.

#### Missingness

Prior work with EMBARC data has reported the availability of fMRI and EEG data [23, 24]. Any missingness from the top associating plasma immune features identified by the predictive power score (PPS) feature selection algorithm, would be imputed using k-nearest neighbors.

### Machine learning (ML) strategy for predicting stage-2 remission

The ML workflow (see Fig. 1A) identified a small set of pre-treatment brain-blood biomarkers associated with baseline depression severity to be used for (a) predicting 16-week outcomes, and (b) serve as inputs to a causal inference algorithm that can match a Stage 1 non-responder to another patient (based on biomarker similarity) who achieved remission to alternative therapies.

#### Feature selection

Feature selection was conducted using the PPS algorithm [25] to identify pre-treatment EEG, fMRI and blood measures associating with baseline HAM-D scores as the target variable. The PPS algorithm identifies and ranks variables based on a data-type agnostic score for linear and non-linear relationships ranging from 0 to 1, with 1 indicating perfect predictive power. The motivation for selecting baseline depression severity as the target variable was due to it often being reported as the top predictor of remission [26]. The algorithm was employed on all participants for each of the modalities who had baseline symptom severity. The top five features as indicated by PPS values (listed in Supplementary Table 3**)** were used to train and test prediction models.

#### Training and external validation cohorts

The dataset was stratified into three independent subgroups, Subgroup A for training prediction models and the Subgroups B & C for external validation of predictive performance (see Fig. 1A). The demographic characteristics of each subgroup is available in Supplementary Table 4. Subgroup A was defined as participants who received sertraline as a treatment at week 16, including sertraline responders and placebo non-responders who switched to sertraline at Stage 2. Subgroup B was defined as participants who were sertraline non-responders and were given bupropion during Stage 2. Subgroup C was defined as placebo responders.

#### Algorithm selection

The goal was to identify a supervised ML algorithm that achieved the best predictive performance when combining all clinical and biological features identified by PPS. The tested suite of algorithms included random forest, K-nearest neighbors, extreme gradient-boosted decision tree-based ensembles (XGBoost), support vector machine (SVM), and penalized regression. Nested cross-validation was used to train and test all algorithms, using 75% of the sample in Subgroup A allocated for training. The training and testing split within Subgroup A was repeated 10 times, and results were averaged over splits. Averaged AUC, accuracy, sensitivity, and specificity for these models are listed in Table 2. The best performing algorithm (defined by highest AUC) was selected to be retrained and retested on other combinations of clinical and biological features (e.g., blood & EEG), with the sample sizes of these available features illustrated in Fig. 1A. This was done to assess the utility of multi-modal integration of features on model performance. Prediction performance (AUC, accuracy, sensitivity, specificity, null information rate) was reported for both training using Subgroup A samples and testing on Subgroup B & C (see Fig. 1B). The null information rate (NIR) is equal to the accuracy of a model that predicts the majority class and represents the null hypothesis for this work. Model-specific variable importance was measured using the ‘vip’ function available in R [27].

**Table 2:** Performance of various classification algorithms on training group and highest performing model tested on cohorts stratified by modalities available.

Comparison of several classification algorithms on training set (Sertraline, *N* = 70)					
	ROC-AUC^a^ [95% CI]	Accuracy	Sensitivity	Specificity	NIR^b^
Random Forest	0.60 [0.39–0.77]	0.58	0.69	0.44	0.57
XGBoost	0.45 [0.36–0.51]	0.46	0.57	0.33	0.57
Support Vector Machines	0.59 [0.32–0.75]	0.54	0.84	0.00	0.57
Penalized Logistic Regression	0.74 [0.64–0.92]	0.69	0.74	0.63	0.57
K- Nearest Neighbors	0.58 [0.42–0.75]	0.59	0.80	0.29	0.57

Highest performing model externally assessed on patient groups with varying levels of data available					
	ROC-AUC [95% CI]	Accuracy	Sensitivity	Specificity	NIR
Clinical (*N* = 197)					
Sertraline (*N* = 119)	0.59 [0.44–0.78]	0.59	0.82	0.23	0.62
Bupropion (*N* = 41)	0.62 [0.50–0.71]	0.60	0.55	0.45	0.63
Placebo (*N* = 37)	0.61[0.42–0.73]	0.66	0.97	0.03	0.84
Clinical + Blood (*N* = 153)					
Sertraline (*N* = 91)	0.71 [0.52–0.88]	0.68	0.83	0.46	0.62
Bupropion (*N* = 30)	0.66 [0.50–0.81]	0.60	0.73	0.40	0.63
Placebo (*N* = 32)	0.67 [0.55–0.77]	0.68	0.82	0.15	0.87
Clinical + Blood + EEG (*N* = 126)					
Sertraline (*N* = 72)	0.63 [0.54–0.74]	0.58	0.64	0.50	0.58
Bupropion (*N* = 27)	0.65 [0.51–0.73]	0.60	0.63	0.44	0.67
Placebo (*N* = 27)	0.70 [0.52–0.81]	0.69	0.79	0.18	0.85
Clinical + Blood + fMRI (*N* = 144)					
Sertraline (*N* = 88)	0.70 [0.56–0.81]	0.62	0.69	0.50	0.60
Bupropion (*N* = 29)	0.70 [0.55–0.84]	0.66	0.56	0.48	0.66
Placebo (*N* = 27)	0.76 [0.65–0.94]	0.72	0.73	0.27	0.89
Clinical + Blood + EEG + fMRI (*N* = 118)					
Sertraline (*N* = 70)	0.74 [0.64–0.92]	0.69	0.74	0.63	0.57
Bupropion (*N* = 24)	0.71[0.59–0.81]	0.64	0.52	0.53	0.70
Placebo (*N* = 24)	0.73 [0.55–0.83]	0.66	0.80	0.13	0.88

^a^ROC-AUC: Receiver Operator Characteristic – Area Under the Curve.

^b^NIR: Null Information Rate.

### Causal inference for facilitating treatment selection

#### Propensity score

**Fig. 2:**
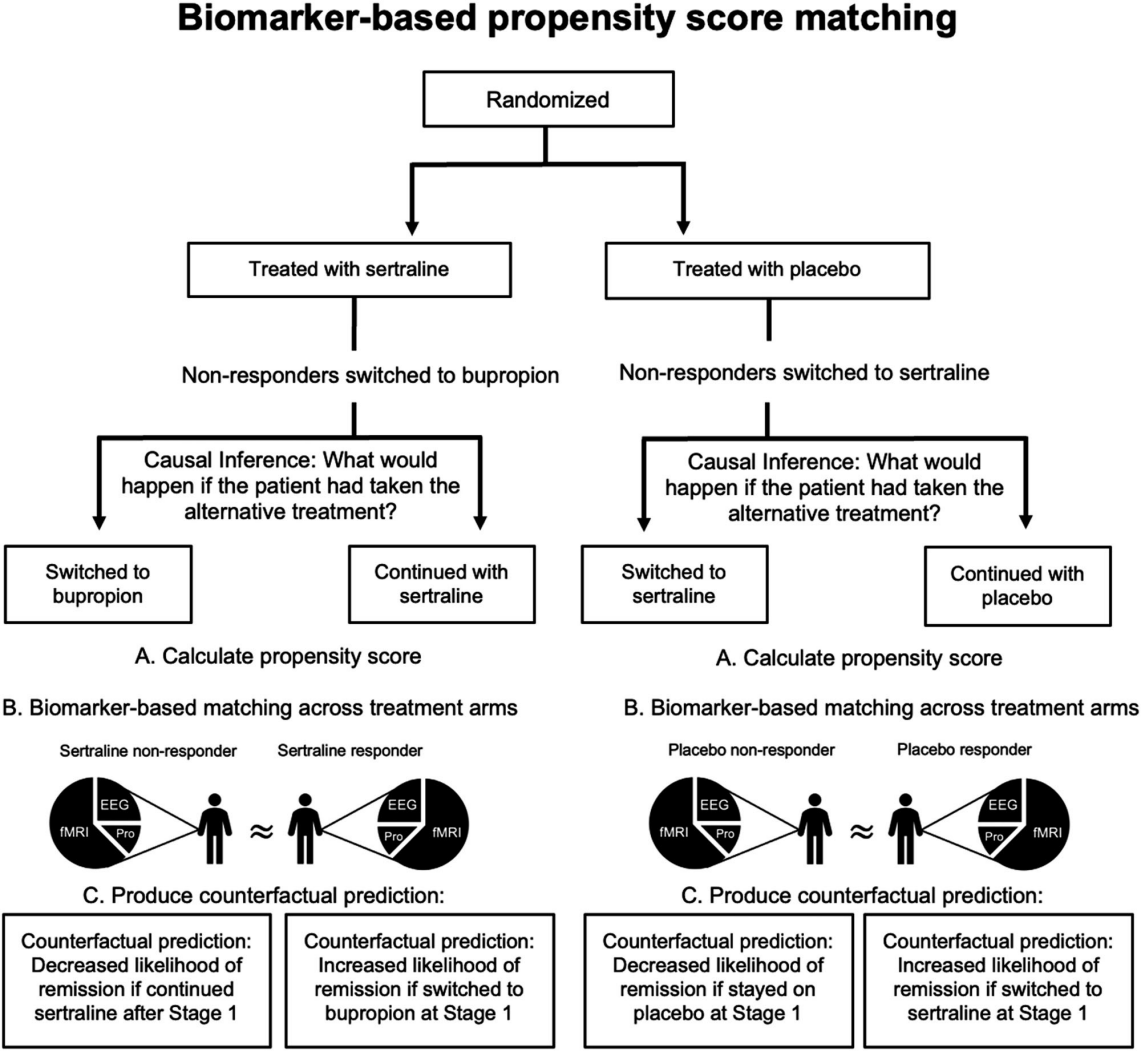
Causal inference pipeline. Pipeline of counterfactual prediction production using biomarker-based propensity score matching. **A** Calculate propensity scores using predictive features. **B** Match participants across Stage 2 arms using propensity scores. **C** Utilize penalized regression to calculate conditional probabilities and produce counterfactual predictions.

The goal of using causal inference methods was to identify a counterfactual (unobserved) hypothesis, i.e., if subjects who could not achieve remission at Stage 2 would have higher likelihood (probability) for an alternative Stage 2 treatment (see Fig. 2). On the placebo arm, probabilities of Stage 2 remission were calculated for placebo and sertraline, and on the sertraline arm, the same probability was calculated for sertraline and bupropion. Propensity score matching was done using the “MatchIt” package in R to match individual participants across Stage 2 treatment arms based on their biological profile (predictive features as determined by supervised ML approach) [28]. It is important to note that biological variables used for matching should not be confused for biomarkers that inform drug mechanism.

#### Generating the counterfactual hypothesis

Causal models were built on both the sertraline arm and placebo arm predicting how subjects would respond on not only the given stage two treatment but on the alternative treatment. After matching, a penalized logistic regression model was used to produce the probability of remitting to both the actual given treatment, and the alternative treatment. In this context, we asked the following questions (see Fig. 2), “would a sertraline non-remitter at Stage 2 have remitted to bupropion had they been switched from sertraline after Stage 1?”, “would a bupropion non-remitter at Stage 2 have eventually remitted from extended exposure to sertraline?”, and “would a non-remitter to placebo at Stage 2 have remitted to sertraline?”

## Results

### Participants and available features

After randomization, of the 296 participants, 278 had fMRI data (7260 features), 212 had immune/metabolic data (50 features), and 275 had EEG data (29,700 features). A total of 197 out of 296 participants had Stage 2 treatment plan and week 16 remission status reported. Out of the 197 participants who had week 16 outcomes, 153 participants had immune/metabolic assays (blood), 144 participants had both immune/metabolic assays and fMRI, 126 participants had immune/metabolic assays and EEG, and 118 participants had immune assays, EEG, and fMRI measures.

### Pre-treatment features for prediction algorithms

Utilizing all available pre-treatment samples for each of the modality of biomarkers (fMRI [brain]: *N* = 278; EEG [brain]: *N* = 275; immune/metabolic [blood]: *N* = 212), top five pre-treatment features associated with baseline MDD were derived using the PPS feature selection algorithm (all features and their predictive power scores are listed in Supplementary Table 3). The top five fMRI features represented connectivity between various brain regions including the anterior cingulate, striatum, calcarine, and more. The EEG features represented alpha band connectivity across different regions. The top five EEG and fMRI network connectivity regions are visualized in Supplementary Fig. 2. The top five blood features were chemokine ligand-1, interleukin-4, interleukin-8, chemokine ligand-26, and chemokine ligand-13. As the final features selected had no missingness across participants, imputation was not needed. These 15 biological pre-treatment features with the addition of 3 demographic features (age, sex, and race) yielded 18 features for predicting remission of MDD at week 16 of treatment.

### Prediction performance

#### In training

Models were trained using data exclusively from Subgroup A. The highest AUC was achieved by a penalized logistic regression integration of demographic, blood, fMRI and EEG brain connectivity measures (mean AUC = 0.74), with sensitivity of 0.74, and specificity of 0.63 (see Table 2). ***In Validation:*** Trained models using data from Subgroup A achieved AUCs of 0.71 and 0.73 respectively using data from Subgroup B and Subgroup C. The receiver operator curve of all performing models is visualized in Fig. 1B. The predictive performance metrics averaged over 10 repeats for all models tested are tabulated in Table 2. ***Top predictors:*** The top predictors of remission at 16 weeks of the fully integrated model were two EEG connectivity features (Schaefer parcel 1 & 98, and Schaefer parcel 26 & 100), two fMRI connectivity features (Schaefer parcel 71 & 52, and Schaefer parcel 97 & 42), and interleukin-8 respectively. Additionally, the top predictors for all combinations of models are summarized in Supplementary Table 5.

### Causal inference for treatment selection hypothesis

The result of propensity score matching was an equal number of responders and non-responders on the Stage 2 arms of the study (48 total in the sertraline arm and 48 in the placebo arm). There were no significant differences between feature values across matched groups (see Supplementary Table 6 for additional results from t-tests). The mean standardized difference in features between the sertraline-to-bupropion group versus the sertraline-to-sertraline group before matching was 17.7% and after matching was 14.2%. The mean standardized difference in covariates between the placebo-to-placebo group versus the placebo-to-sertraline group before matching was 43.4% and after matching was 24.6%. Ideally the mean standardized differences would be lower, however, after matching both groups are under the 25% cutoff that is deemed adequate for balancing [29].

Penalized logistic regression results which produced averaged counterfactual probabilities by treatment and observed outcome are present in Table 3, and probabilities for each individual subject are in Supplementary Table 7. Within the placebo arm, one of the three placebo non-remitters had a higher likelihood of remitting to sertraline. Within the sertraline arm, two of the eight sertraline non-remitters had a higher likelihood of remitting to bupropion, and all bupropion non-remitters had a higher likelihood of remitting to sertraline (indicating that these participants were likely to remit had they continued sertraline treatment for a longer duration). Within the sertraline arm, three non-remitting subjects had low probability ( < 50%) of remitting to either pharmacotherapy (sertraline or bupropion). Factual outcomes (i.e., those observed in the study) were predicted with 82% accuracy.

**Table 3:** Counterfactual predictions by treatment arm and observed outcome.

Placebo Arm			
Stage 2 treatment	Observed Outcome	Mean prediction of remission if patient treated with Sertraline	Mean prediction of remission if patient remained on Placebo
Placebo	Non-remitters	0.42*	0.47
Placebo	Remitters	0.52*	0.93
Sertraline	Non-remitters	0.40	0.83*
Sertraline	Remitters	0.60	0.90*

Sertraline Arm			
Stage 2 treatment	Observed Outcome	Mean prediction of remission if patient remained on Sertraline	Mean prediction of remission if patient treated with Bupropion
Sertraline	Non-remitters	0.55	0.35*
Sertraline	Remitters	0.72	0.29*
Bupropion	Non-remitters	0.64*	0.25
Bupropion	Remitters	0.57*	0.39

^*^Indicates counterfactual prediction.

## Discussion

Antidepressant treatment selection continues to be “*artisanal*” in that treatment management is a “try-and-try-again” process depending on treatment outcomes in sequential pharmacotherapy [30]. Although ML algorithms show promise in establishing predictability of antidepressant response [14], there is a need to extend the analytical capabilities with biomarkers toward facilitating antidepressant selection. The work presented herein comprises a strong basis for future research efforts aimed at utilizing predictive biomarkers for developing treatment selection models for MDD—work that is crucial for precision psychiatry. To do so, validation of biomarkers will be needed, involving studies evaluating the efficacy of AI-guided treatment selection v. standard of care. This report addresses this critical need towards precision psychiatry of major depressive disorder and as a novel application of casual inference approaches [31], by integrating brain and blood biomarkers for generating counterfactual hypothesis of potential remission (or lack of) to alternative treatments using sequential treatment data from the EMBARC study.

Prior work by Iniesta et al. identified that prediction performance of AUC > 0.70 is considered clinically meaningful [32]. In this context, this report found that integrating blood with fMRI or fMRI and EEG achieved AUC > 0.70 in all subgroups both during training and testing prediction of sequential treatment outcomes. Ultimately, predicting outcomes to specific treatments serves as a first step towards developing effective treatment selection tools, and more work is being done to identify how to best select first and second-line treatments. Data from the STAR-D trial, demonstrated that several clinical features are predictive of whether patients will respond to second line antidepressants [33]. A recent analysis created the first pharmacological differential treatment benefit model for MDD by using clinical and demographic data from 17 studies to predict remission and generate remission probabilities for five other treatments [34]. Other work has used ML to create a tool that assists clinicians in deciding between esketamine nasal spray and transcranial magnetic stimulation for treatment resistant depression [35]. This report extends the literature of antidepressant response predictions, by also utilizing predictive biomarkers for potentially aiding in treatment selection. Finally, this report encourages future research to optimize the selection of predictive biomarkers that can be easily obtained in clinical practice.

The top predictor of the model was connectivity between the left fusiform of the visual network, and the right middle frontal of the default mode network at the alpha frequency (Schaefer parcel 1 & 98). The default mode network (DMN) is believed to be activated during internal mental simulations such as thinking of one’s past or worrying about the future [36]. Previous work has shown that there is a significant difference in DMN activity between healthy control groups and individuals with MDD [37]. In a study attempting to uncover suicide-risk specific features from fMRI connectivity analysis, it was found that increased suicide attempts had an increased connectivity within the visual network which extended to regions of the DMN [38]. The DMN has shown up as a signature that is predictive of depressive symptom improvement following ECT, and treatment response to escitalopram [39, 40] Future exploration of the utility of this marker as a prognostic biomarker of treatment outcomes across subsets of patients who differ by baseline severity is warranted.

The top blood-based, and fifth overall predictor was interleukin (IL)-8. Observational studies demonstrated that IL-8 are found in higher levels of patients with lower depressive symptom severity with treatment resistant depression [41]. While individual studies highlighted a difference in IL-8 levels between patients with and without MDD, meta-analyses have found inconclusive results [42]. These analyses have shown that individuals with MDD don’t have differing levels of IL-8 compared to those without, however a limitation is the size of these studies [43, 44]. Despite this, IL-8 has previously been discussed in the literature to be predictive of depression response to ketamine, electroconvulsive therapy, and paroxetine [45, 46]. While the role IL-8 role plays in MDD is still not clear, prior research supports the assumption that IL-8 can be predictive of treatment response.

### Limitations

Despite demonstrating cross-arm replication in predictive performance, there is no independent study with EEG, fMRI and blood data to validate the predictive performance and counterfactual hypothesis. Even with efforts to reduce the chance of overfitting through cross-validation and external replication across EMBARC study arms, future work with replications across studies are needed for establishing reliability of prediction models. Of the 197 subjects used to build and test the models, 38.6% were male, which is characteristic of male incident cases of MDD globally (37.7%) [47]. Additionally, several algorithms were only tested on the sample of participants with all possible biological data collected, and future efforts are needed to collect biological measures on larger cohorts with greater representation of ethnic populations that may facilitate generalization of biomarker-guided treatment selection models.

Finally, causal inference and counterfactual prediction is an evolving field of analyses in individualizing antidepressant response. Full verification of the validity and completeness of the model is not possible without observable data; however, there are aspects of the causal model which are empirically testable, such as the accuracy of predicting observed outcomes [48]. It is important to note that biological variables used for propensity-score matching should not be confused for biomarkers that inform antidepressant response mechanism. Additional efforts are needed to prospectively evaluate counterfactual models, rather than solely calculating error on factual outcomes. While ML formulations offer limited interpretability by design, they offer more insight into predictability of treatment outcomes and rank-order predictor variables. When interpreting predictions, variable importance is included which provides a rank order list of variables that contribute the most when making predictions. Although the machine learning approach in this work identified that combining biomarkers of multiple modalities increases predictability of antidepressant remission and aids in treatment selection, future work is needed to elucidate the mechanistic effects of these interactions in the context of precision medicine for MDD.

## Conclusions

This work demonstrates that a combination of EEG, fMRI, and plasma-derived immune markers can be important prognostic, integrative indicators to identify patients who are likely to experience remission in following the antidepressant therapies studied in EMBARC. The model trained on sertraline-treated patients performed well on additional treatment subgroups within EMBARC, suggesting that the model may predict treatment response more generally and might not be useful in selecting specific treatments. A causal model with these biomarkers was constructed, providing estimations of the likelihood of an individual remitting to their assigned Stage 2 treatment, as well as the alternative treatment. The model predicted factual (observed) events with high accuracy, and generated counterfactual predictions that can be validated in future studies. Computed estimates from the model demonstrated that some subjects had a low probability of achieving remission on either sertraline or bupropion, potentially identifying subjects who may benefit from more intensive intervention or monitoring.

## Supplementary information

Supplemental Table 7

Supplemental Materials

## Data Availability

Data and code are available upon reasonable request to Dr. Madhukar H. Trivedi.

## Prior Presentation

This work’s earlier findings were presented as a Poster at the 2025 Annual meeting of Am. College of Neuropsychopharmacology, Scottsdale, AZ, USA.
