# Supplementary material for: Towards causal inference-based antidepressant selection with brain and blood biomarkers
Source: Neuropsychopharmacology. 2025 Sep 5;51(3):622–30. doi: 10.1038/s41386-025-02183-3 (PMC12823655; doi:10.1038/s41386-025-02183-3)
Supplement: Supplementary file 2 — Supplemental Materials [file 41386_2025_2183_MOESM2_ESM.pdf]

Supplementary Figures

Supplementary Figure 1. Analyte concentrations pre- and post-ComBat correction

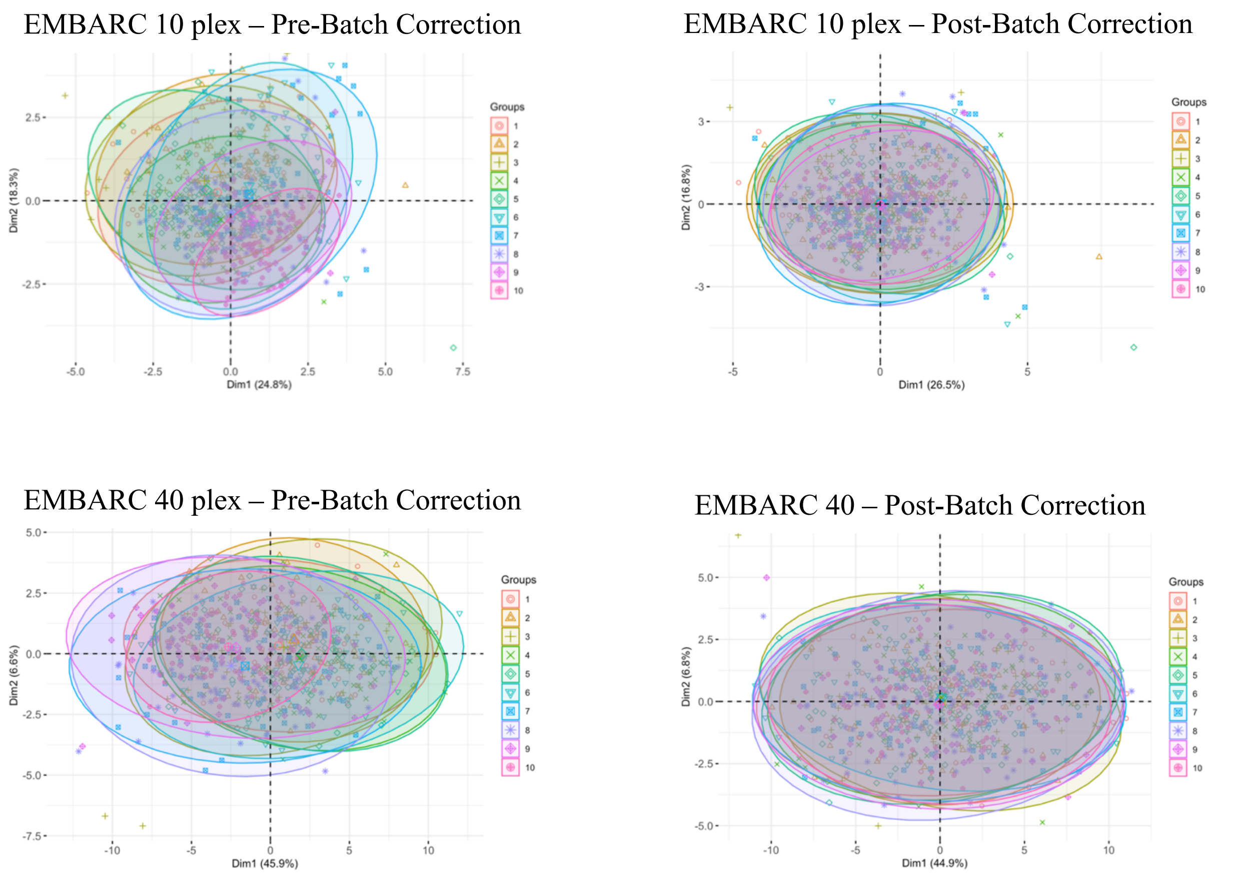

Upper panels show the distribution of analyte concentrations measured using the 10 plex panel pre (left upper) and post (right upper) batch correction. Individual points represent each sample correction, coloured by plate. Lower panels show the distribution of analyte concentrations measured using the 40 plex panel pre (bottom left) and post (bottom right) batch correction.

Supplementary Figure 2. Brain regions visualized of top five fMRI and EEG features

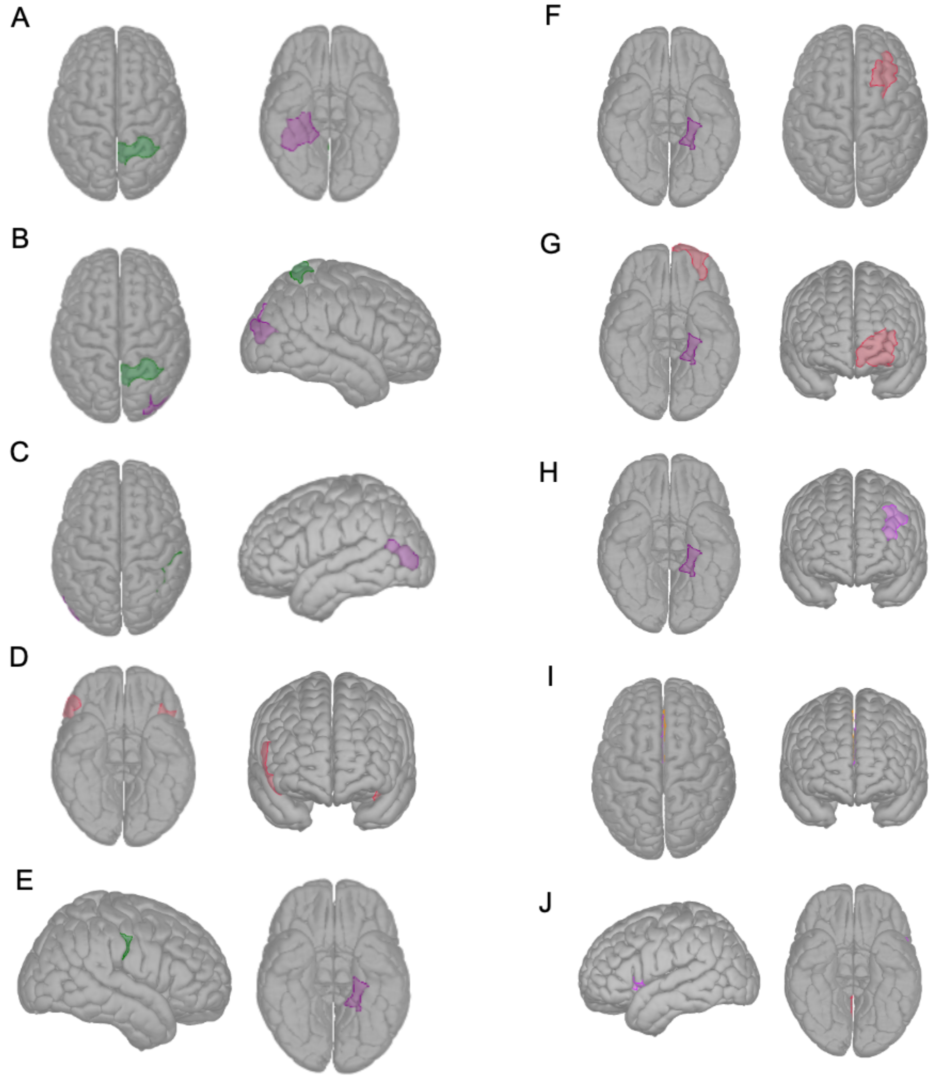

(A-E) fMRI brain connectivity:
(A) Right precuneus of sensorimotor network (green) and right fusiform of visual network (purple). (B) Right precuneus of sensorimotor network (green) and right middle occipital of visual network (purple). (C) Right inferior parietal of sensorimotor network (green) and left middle temporal of visual network (purple). (D) Right superior medial frontal of default mode network (red) and left inferior orbitofrontal of default mode network (red). (E) Right postcentral of the sensorimotor network (green) and left fusiform of the visual network (purple).
(F-J) EEG brain connectivity:
(F) Left fusiform of the visual network (purple), and right middle frontal of the default network (red). (G) Left fusiform of visual network (purple) and left middle frontal of the default mode network (red). (H) Left fusiform of visual network (purple) and left middle frontal of the salience network (magenta). (I) Right middle cingulate of the executive control network (orange) and left anterior cingulate of the salience network (magenta). (J) Left anterior insula of the salience network (magenta) and right posterior cingulate of the default mode network (red).

Supplementary Tables

Supplementary Table 1. Summary of plasma analyte levels (pg/ml) measured using the Bio-Plex Human Diabetes 10-Plex assay

	Hu C-peptide	Hu Ghrelin	Hu GIP	Hu GLP-1	Hu Glucagon	Hu Insulin	Hu Leptin	Hu PAI-1	Hu Resistin	Hu Visfatin
Mean	634.7	412.8	698.5	189.8	1178.3	507.1	11532.6	11160.2	4754.6	3159.4
SD	442.79	257.04	1276.11	84.27	300.44	678.28	9695.63	7787.28	2221.81	8378.43
Min Value	1.2	66.7	6.2	44.3	665.8	0.6	56.2	1667.0	1137.8	254.0
Max Value	3060.8	1681.1	24587.6	1308.6	4987.2	6918.6	64043.1	70676.5	14435.3	119425.4
% below LOD	2.62	0	1.05	0.13	0	0.26	0.79	0	0	0.26

Abbreviation: LOD – limit of detection

Supplementary Table 2. Summary of plasma analyte levels (pg/ml) measured using the Bio-Plex

	6Ckine/CCL21	BCA-1/CXCL13	CTACK/CCL27	ENA-78/CXCL5	Eotaxin/CCL11	Eotaxin-2/CCL24	Eotaxin-3/CCL26	Fractalkine/CX3CL1	GCP-2/CXCL6	GM-CSF
Mean	4662.91	22.31	788.01	429.34	29.76	225.92	20.74	171.3	36.17	41.89
SD	1927.91	22.63	349.29	311.39	10.19	183.9	11.3	121.02	24.98	38.04
Min	497.22	6.06	110.27	13.42	11.98	29.72	2.33	43.12	3.65	0.64
Max	14929.6	300.39	2518.43	3524.12	99.71	1451.24	94.32	2000.09	193.31	287.63
% below LOD	0	0	0	1.96	0	0	0.13	0	0	8.9

	Gro-a/CXCL1	Gro-b/CXCL2	I-309/CCL1	IFN-y	IL-1b	IL-2	IL-4	IL-6	IL-8/CXCL8	IL-10
Mean	171.61	321.27	39.14	41.83	2.58	12.98	20.3	16.39	7.43	18.97
SD	101.03	297.46	10.88	26.46	1.19	6.87	10.99	10.98	4.54	11.7
Min	46.29	14.65	15.6	7.91	0.52	2.83	2.09	1.36	2.07	3.09
Max	1117.41	2548.37	84.34	231.49	14.79	48.98	75.53	72.7	71.38	77.79
% below LOD	0	0	0	0	0	0	0.39	0	0	0

	IL-16	IP-10/CXCL1	I-TAC/CXCL11	MCP-1/CCL2	MCP-2/CCL8	MCP-3/CCL7	MCP-4/CCL13	MDC/CCL22	MIF	MIG/CXCL9
Mean	623.58	155.16	25.81	27.69	24.8	71.25	58.41	610.84	8228.43	163.06
SD	256.41	109.83	23.12	8.71	14.18	36.5	39.97	232.52	8561.33	178.63
Min	91.52	25.06	3.33	10.63	4.66	14.19	10.64	55.73	218.06	29.25
Max	1844.27	1151.46	178.18	77.07	189.5	259.09	274.5	1825.56	68489.63	2613.09
% below LOD	0	0	0	0	0	0	0	0	0	0

	MIP-1a/CCL3	MIP-1d/CCL15	MIP-3a/CCL20	MIP-3b/CCL19	MPIF-1/CCL23	SCYB16/CXCL16	SDF-1a+b/CXCL12	TARC/CCL17	TECK/CCL25	TNF-a
Mean	12.4	2735.11	10.46	124.09	290.5	496.61	1274.77	50.61	346.91	21.68
SD	42.79	932.62	8.19	71.86	194.08	158.02	389.24	47.79	152.1	10.28
Min	2.17	545.46	2.18	18.25	8.1	70.19	211.37	2.9	99.08	3.39
Max	716.89	7084.55	66.44	572.73	1137.62	1187.81	2617.42	389.18	1365.57	73.66
% below LOD	0	0	0	0	0	0	0	0	0	1.44

Supplementary Table 3. Features as selected by predictive power scores

Features	Predictive Power Score
**fMRI**	
Connectivity between Schaefer parcel 71 & 52	0.04
Connectivity between Schaefer parcel 71 & 57	0.04
Connectivity between Schaefer parcel 69 & 7	0.03
Connectivity between Schaefer parcel 97 & 42	0.03
Connectivity between Schaefer parcel 68 & 1	0.03
**EEG**	
Connectivity between Schaefer parcel 1 & 98 at the alpha frequency	0.01
Connectivity between Schaefer parcel 1 & 45 at the alpha frequency	0.01
Connectivity between Schaefer parcel 1 & 27 at the alpha frequency	0.01
Connectivity between Schaefer parcel 87 & 28 at the alpha frequency	0.01
Connectivity between Schaefer parcel 26 & 100 at the alpha frequency	0.01
**Blood**	
Chemokine ligand-1	0.05
Interleukin-4	0.04
Interleukin-8	0.03
Chemokine ligand-26	0.03
Chemokine ligand-13	0.03

Supplementary Table 4. Demographic characteristics by subgroup

	Subgroup A (N=119)	Subgroup B (N=41)	Subgroup C (N=37)	Total (N=197)
**Age**				
Mean (SD)	39.1 (13.4)	36.6 (14.5)	36.2 (12.2)	38.0 (13.4)
Median [Min, Max]	38.0 [18.0, 65.0]	30.0 [18.0, 65.0]	32.0 [19.0, 63.0]	36.0 [18.0, 65.0]
**Sex**				
Female	72 (60.5%)	28 (68.3%)	21 (56.8%)	121 (61.4%)
Male	47 (39.5%)	13 (31.7%)	16 (43.2%)	76 (38.6%)
**Race**				
American Indian or Alaska Native	0 (0%)	0 (0%)	1 (2.7%)	1 (0.5%)
Asian	4 (3.4%)	3 (7.3%)	4 (10.8%)	11 (5.6%)
Black	19 (16.0%)	10 (24.4%)	5 (13.5%)	34 (17.3%)
Native Hawaiian	0 (0%)	0 (0%)	0 (0%)	0 (0%)
White	85 (71.4%)	25 (61.0%)	26 (70.3%)	136 (69.0%)
Other	11 (9.2%)	3 (7.3%)	1 (2.7%)	15 (7.6%)

Supplementary Table 4. Subgroup A is defined as placebo non-responders and sertraline responders. Subgroup B is defined as sertraline non-responders who were switched to bupropion in Stage 2. Subgroup C is defined as placebo responders.

Supplementary Table 5. Top Predictors Across Model Combinations

Model	Top 5 Predictors of Stage 2 (16 week) remission
**Clinical + Blood**	
	Race
	Chemokine Ligand-1
	Sex
	Chemokine Ligand-13
	Interleukin-8
**Clinical + Blood + EEG**	
	Race
	Interleukin-8
	Chemokine Ligand-1
	EEG Connectivity between Schaefer Parcel 1 & 45
	Interleukin-4
**Clinical + Blood + fMRI**	
	Race
	Interleukin-8
	Sex
	Chemokine ligand-13
	fMRI Connectivity between Schaefer Parcel 67 & 68
**Clinical + Blood + EEG + fMRI**	
	EEG Connectivity between Schaefer Parcel 1 & 98
	EEG Connectivity between Schaefer Parcel 26 & 100
	fMRI Connectivity between Schaefer Parcel 71 & 52
	fMRI Connectivity between Schaefer Parcel 97 & 42
	Interleukin-8

Supplementary Table 6. Mean values of features after propensity score matching

	Placebo Arm			Sertraline Arm		
**Features**	**Mean of participants taking Placebo**	**Mean of participants taking Sertraline**	**p-value**	**Mean of participants taking Sertraline**	**Mean of participants taking Bupropion**	**p-value**
**fMRI**						
Connectivity between Schaefer parcel 71 & 52	0.15	0.21	0.47	0.13	0.13	0.99
Connectivity between Schaefer parcel 71 & 57	0.21	0.24	0.72	0.21	0.17	0.62
Connectivity between Schaefer parcel 69 & 7	0.14	0.15	0.98	0.09	0.12	0.73
Connectivity between Schaefer parcel 97 & 42	0.29	0.29	0.99	0.25	0.15	0.10
Connectivity between Schaefer parcel 68 & 1	0.04	0.10	0.29	0.02	0.06	0.18
**EEG**						
Connectivity between Schaefer parcel 1 & 98 at the alpha frequency	0.15	0.11	0.06	0.10	0.11	0.69
Connectivity between Schaefer parcel 1 & 45 at the alpha frequency	0.15	0.12	0.16	0.11	0.11	0.97
Connectivity between Schaefer parcel 1 & 27 at the alpha frequency	0.15	0.11	0.05	0.10	0.11	0.91
Connectivity between Schaefer parcel 87 & 28 at the alpha frequency	0.16	0.12	0.07	0.13	0.11	0.40
Connectivity between Schaefer parcel 26 & 100 at the alpha frequency	0.17	0.14	0.13	0.12	0.11	0.71
**Blood**						
Chemokine ligand-1	6.94	7.09	0.38	7.30	7.37	0.70
Interleukin-4	3.95	3.97	0.94	4.15	4.31	0.57
Interleukin-8	2.42	2.52	0.50	4.27	4.36	0.67
Chemokine ligand-26	3.93	4.04	0.57	2.80	2.94	0.40
Chemokine ligand-13	5.22	5.26	0.86	5.78	5.78	0.98

Supplementary Text

Supplemental Methods

Functional Magnetic Resonance Imaging (fMRI): MRI scans were collected on 3 Tesla MR systems at all EMBARC sites. The resting state functional MRI (rsfMRI) acquisition parameters across the four sites were similar: single-shot gradient echo EPI, TR = 2000 ms, TE = 28ms, voxel size = 3.2 X 3.2 X 3.1 mm^3, 39 axial slices, 180 image volumes, and duration of 6 minutes. The high resolution T_1-weighted image parameters were: 160 sagittal slices, voxel size = 1 X 1 X 1 mm^3, and FOV = 256 X 256 X 160mm^3.

The rsfMRI data were processed using the Connectivity Toolbox [1] and SPM 8. Briefly, the data were preprocessed with a standard pipeline, namely slice time correction, motion corrected (realignment and unwarp), spatial normalization, and smooth with an 8mm Gaussian Kernel. Scrubbing was performed with the Artifact Detection Toolbox [2] using the following criteria: normalized global BOLD signal $Z \geq 3.0$ and subject motion threshold ≥ 0.5. Physiological and other spurious sources of noise were estimated using the aCompCor method and used as covariates [3]. Lastly, the residual BOLD time series was band-pass filtered using $0.009\text{Hz} < f < 0.08\text{Hz}$ to only keep the appropriate frequency fluctuations.

Seed-based functional connectivity was computed using the 121 brain regions, namely 100-brain region parcellation [4], hippocampus [5], ventral striatum [6], thalamus, and amygdala [7]. The 100 brain parcellation was divided into the seven major networks, default mode, salience, executive control, dorsal attention, somatomotor, limbic, and visual networks.

EEG Preprocessing

The resting state paradigms were acquired in four 2-minute blocks, in the following sequence: eyes open, closed, closed, open. This report only investigated the eyes open condition as it is the paradigm more translatable to clinical practice (paradigm used in rsFMRI and it enables the operator to ensure patients are not asleep). Acquired data were down sampled to 250 Hz, high-pass filtered at 1 Hz, notch filtered at 60 Hz and its harmonic, and low-pass filtered at 30 Hz, and artifactual channels interpolated. This frequency range was selected as suggested by previous work demonstrating both that the majority of connectivity features emerge at and below the beta frequency band and that data are more susceptible to muscle artifacts at higher

frequencies [8]. Source localization was computed using the Brainstorm toolbox [9]. A 3-layer symmetric boundary was calculated using the OpenMEEG plugin, and rotating dipoles were generated [10]. An unconstrained imaging kernel mapping the channel-space EEG to source-space current density was calculated. EEG data was then convolved with a complex-valued Morlet wavelet to obtain the theta, alpha, and low beta frequency bands. For each frequency band, the unconstrained imaging was reduced and later multiplied with the channel-space analytic signal to produce the source-space analytic signal. Multiplying these source-space data by its complex conjugate resulted in plan power envelopes. Vertex-wise global connectivity between plan and orthogonalized power envelopes were computed. The final representative connectivity measure was the median connectivity value across windows.

These matrices are directional; that is, vertex 1 orthogonalized with respect to vertex 2 is not the same as vertex 2 orthogonalized with respect to vertex 1. These data were then inspected for quality assurance by comparing global connectivity measures to those found in separate studies [8, 11, 12] and agreement was verified. Connectivity measures were then recomputed using a narrow bandwidth of 1 Hz at center frequencies 2-28 Hz. These data were used to produce frequency-connectivity curves at 3 landmark early sensory areas (visual, auditory, and somatosensory) to verify congruence with established sensory connectivity curves[8]. The connectivity matrices were then used to calculate connectivity hubs.

Mapping constituent vertices to their respective networks is necessary when producing network connectivity inferences. Yeo's seminal 2011 study of 1000 HCs is a standard of fMRI atlases [13]. In this report, the 100 parcel, 7 network Schaefer parcellation to the cortical tessellation was used. These parcels were used to classify the emergent connectivity hubs network membership and is consistent with the ROI used for rsfMRI in this report.

Blood Analyte Preprocessing

Plasma was extracted, aliquoted, frozen, and shipped to UT Southwestern, Dallas, TX, and stored at -80C until processing. Plasma samples were analysed using the Bio-Plex Human Diabetes 10-Plex assay and using the Bio-Plex Human Chemokine Panel, 40 Plex (Bio-Rad Laboratories, Hercules, CA, USA). Immunoassays were performed at the Microarray Core at UT Southwestern Medical Center with a Bioplex 200 instrument that was equipped with Bioplex Manager software, version 6.0 (Bio-Rad Laboratories, Hercules, CA, USA). Protein levels were calculated in pg/mL using the standards provide in the kit. For samples where the concentration was below the limit of

detection (LOD), the lowest detected value (standard or sample) identified for that analyte in that plate was used. Data processing of raw concentration data was performed using R version 4.3.2. Sample concentration data were log2 normalized. To control for between plate variance, the ComBat function in the *sva* package (v 3.50.0) was used to remove batch effects. Summary concentration values for all analytes are provided in **Supplementary Table 1 and 2**. Principal component analysis was used to visualize the plates pre- and post-Combat batch correction **Supplementary Figure 1**. Log2, batch corrected analyte levels were used in the downstream analyses.

Analytes in the 10-Plex assay included C-peptide, ghrelin, gastric inhibitory polypeptide (GIP), glucagon-like peptide-1 (GLP-1), insulin, leptin, plasminogen activator inhibitor-1 (PAI-1), resistin, and visfatin. Analytes in the 40-Plex panel included chemokine ligand (CCL) 21(6Ckine/CCL21), cutaneous T cell-attracting chemokine (CTACK/CCL27), eotaxin (CCL11), eotaxin-2 (CCL24), eotaxin-3 (CCL26), CCL1/I-309, monocyte chmotactic protein (MCP)-2 (CCL2), MCP-2/CCL8, MCP-3/CCL7, MCP4/CCL13, macrophage-derived chemokine (MDC/CCL22), macrophage inhibitory protein (MIP)-1α (CCL3), MIP-1δ (CCL15), MIP-3α (CCL20), MIP-3β (CCL19), myeloid progenitory inhibitory factor (MPIF/CCL23), thymus and activation-regulation chemokine (TARC/CCL17), thymus-expressed chemokine (TECK/CCL25), B cell-attractant chemokine 1 (BCA-1) also called C-X-C motif chemokine 13 (CXCL13), epithelial neutrophil-activating peptide 78 (ENA-78/CXCL5), fractalkine (CX3CL1), interferon-gamma-inducible protein 10 (IP-10/CXCL10), interferon-inducible T cell alpha chemoattractant (I-TAC), granulocyte chemotactic protein (GCP) 2 (CXCL6), MIG/CXCL9, SCYB16/CXCL16, stromal cell-derived factor 1 (SDF-1/CXCL12), granulocyte-macrophage colony-stimulating factor (GM-CSF), Gro-α/CXCL1, Gro-β/CXCL2, interferon-γ (IFN-γ), interleukin (IL)-1β, IL-2, IL-4, IL-6, IL-8/CXCL8, IL-10, IL-16, macrophage migration inhibitory factor (MIF), and tumor necrosis factor (TNF)-α.

Supplementary References

1. Whitfield-Gabrieli, S. and A. Nieto-Castanon, *Conn: a functional connectivity toolbox for correlated and anticorrelated brain networks.* Brain Connectivity, 2012. **2**: p. 125-141.
2. Mazaika, P., S. Whitfield-Gabrieli, and J.C. Cooper, *Detection and repair of transient artifacts in fMRI data.* Human Brain Mapping, 2005. **26**: p. S36.
3. Muschelli, J., et al., *Reduction of motion-related artifacts in resting state fMRI using aCompCor.* Neuroimage, 2014. **96**: p. 22-35.
4. Schaefer, A., et al., *Local-Global Parcellation of the Human Cerebral Cortex From Intrinsic Functional Connectivity MRI.* bioRxiv, 2017: p. 135632.
5. Chen, A.C. and A. Etkin, *Hippocampal network connectivity and activation differentiates post-traumatic stress disorder from generalized anxiety disorder.* Neuropsychopharmacology, 2013. **38**(10): p. 1889-98.
6. Choi, E.Y., B.T. Yeo, and R.L. Buckner, *The organization of the human striatum estimated by intrinsic functional connectivity.* Journal of neurophysiology, 2012. **108**(8): p. 2242-2263.
7. Etkin, A., et al., *Failure of anterior cingulate activation and connectivity with the amygdala during implicit regulation of emotional processing in generalized anxiety disorder.* Am J Psychiatry, 2010. **167**(5): p. 545-54.
8. Hipp, J.F., et al., *Large-scale cortical correlation structure of spontaneous oscillatory activity.* Nat Neurosci, 2012. **15**(6): p. 884-90.
9. Tadel, F., et al., *Brainstorm: a user-friendly application for MEG/EEG analysis.* Comput Intell Neurosci, 2011. **2011**: p. 879716.
10. Gramfort, A., et al., *OpenMEEG: opensource software for quasistatic bioelectromagnetics.* Biomed Eng Online, 2010. **9**: p. 45.
11. Hipp, J.F. and M. Siegel, *BOLD fMRI Correlation Reflects Frequency-Specific Neuronal Correlation.* Curr Biol, 2015. **25**(10): p. 1368-74.
12. Toll, R.T., et al., *An Electroencephalography Connectomic Profile of Posttraumatic Stress Disorder.* Am J Psychiatry, 2020. **177**(3): p. 233-243.
13. Yeo, B.T., et al., *Estimates of segregation and overlap of functional connectivity networks in the human cerebral cortex.* Neuroimage, 2014. **88**: p. 212-27.
